# Improved Topical Drug Delivery: Role of Permeation Enhancers and Advanced Approaches

**DOI:** 10.3390/pharmaceutics14122818

**Published:** 2022-12-15

**Authors:** Victor Hmingthansanga, Nidhi Singh, Superna Banerjee, Sivakumar Manickam, Ravichandiran Velayutham, Subramanian Natesan

**Affiliations:** 1 Department of Pharmaceutics, Advanced Formulation Laboratory, National Institute of Pharmaceutical Education and Research Kolkata, Chunilal Bhawan, 168, Maniktala Main Road, Kolkata 700054, India; 2 Petroleum and Chemical Engineering, Faculty of Engineering, Universiti Teknologi Brunei, Bandar Seri Begawan BE1410, Brunei; 3 National Institute of Pharmaceutical Education and Research Kolkata, Kolkata 700054, India

**Keywords:** transdermal, drug delivery, stratum corneum, permeation enhancers, ionic liquids, cell penetration peptides, microneedle

## Abstract

The delivery of drugs via transdermal routes is an attractive approach due to ease of administration, bypassing of the first-pass metabolism, and the large skin surface area. However, a major drawback is an inability to surmount the skin’s stratum corneum (SC) layer. Therefore, techniques reversibly modifying the stratum corneum have been a classical approach. Surmounting the significant barrier properties of the skin in a well-organised, momentary, and harmless approach is still challenging. Chemical permeation enhancers (CPEs) with higher activity are associated with certain side effects restricting their advancement in transdermal drug delivery. Furthermore, complexity in the interaction of CPEs with the skin has led to difficulty in elucidating the mechanism of action. Nevertheless, CPEs-aided transdermal drug delivery will accomplish its full potential due to advancements in analytical techniques, synthetic chemistry, and combinatorial studies. This review focused on techniques such as drug–vehicle interaction, vesicles and their analogues, and novel CPEs such as lipid synthesis inhibitors (LSIs), cell-penetrating peptides (CPPs), and ionic liquids (ILs). In addition, different types of microneedles, including 3D-printed microneedles, have been focused on in this review.

## 1. Introduction

Discovering a novel drug is an exceptionally costly and time-consuming activity. Nevertheless, restructuring the modules and ways to ferry the drug into the systemic circulation is a less challenging and prolific task. If each drug is delivered in the most favourable and ideal approach to an individual patient, the engineering of the dosage form to deliver the precise amount of the drug at the precise time to the exact target position becomes complicated. If discharged slowly from the dosage form, the administered drugs will probably not be absorbed and not enter the systemic circulation. If the drug is discharged too quickly from the dosage form, the patient may experience inconvenient effects, and its looked-for effect may not last as long as required. The solution to this problem is the development of transdermal drug delivery systems (TDDSs), which can ferry the drug through the portals of the skin and uphold clinically effective concentrations over an extended period.

TDDSs can offer numerous advantages over conventional ones, including improved efficacy, safety, avoiding first-pass metabolism, greater convenience, and better patient compliance. By delivering a steady stream of the drug into the systemic circulation over an extended period, the transdermal drug delivery system can circumvent the “peak and valley”, which is an intrinsic consequence of oral or injectable dosage form and can facilitate more controlled, effective treatment. The main limitations of the TDDSs are predominantly associated with the skin’s barrier function. The skin is a multi-laminate tissue; the outermost layer comprises the major barrier to drug permeation. A unique hierarchical structure of lipid-rich matrix with embedded corneocytes in the upper strata (15 μm) of skin, the stratum corneum (SC), is responsible for this barrier and severely constrains the absolute amount of a drug that is absorbed across a reasonable area of the skin during a dosing period. The physicochemical properties of the drug, such as molecular weight, log partition coefficient (log P), degree of ionisation, and melting point, also affect the transdermal permeation. Drugs for which transdermal administration would be beneficial clinically do not possess the required physicochemical properties to penetrate the viable skin. Therefore, the minimum requirements for a drug to penetrate the skin are as follows: (1) high potency (dose < 10 mg/day), (2) small molar mass (molar mass < 500 g/mol), (3) log P (moderate 1–5), and (4) melting point (<250 °C). These physicochemical properties are the general recommendation, not an established one. Researchers have developed several strategies and methods for enhancing transdermal drug delivery across the skin based on these factors. Strategies such as modifying SC and its related methods have gained huge interest due to their various advantages.

This review focuses on techniques such as drug–vehicle interaction, vesicles and their analogues, and chemical permeation enhancers (CPEs). In addition, it focuses on novel CPEs such as lipid synthesis inhibitors (LSIs), cell-penetrating peptides (CPPs), ionic liquids (ILs), and an ideal approach for the selection of CPEs. Moreover, it emphasises different types of microneedles, including 3D-printed microneedles for transdermal drug delivery.

The literature search for this review paper was completed on PubMed (https://pubmed.ncbi.nlm.nih.gov/, accessed on 16 November 2020), PubChem (https://pubchem.ncbi.nlm.nih.gov/, accessed on 20 November 2020), and Google Scholar (https://scholar.google.com/, accessed on 18 November 2020) by entering keywords such as transdermal drug delivery, chemical permeation enhancers, liposomes, and microneedles. 

## 2. Structure of the Skin 

**Figure 1:**
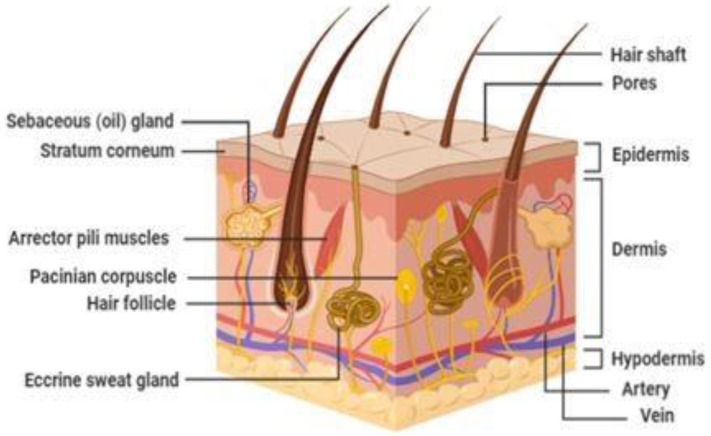
Microscopic structure of the skin.

The skin may be a major barrier to the transdermal delivery of drugs. On the other hand, it may also be highly regarded for its protective and self-healing abilities [1]. The skin covers around 2 m^2^ of an average adult, preventing water loss from our body and shielding us from undesired external influences [2]. A schematic representation of the skin is shown in Figure 1.

### 2.1. Epidermis

**Figure 2:**
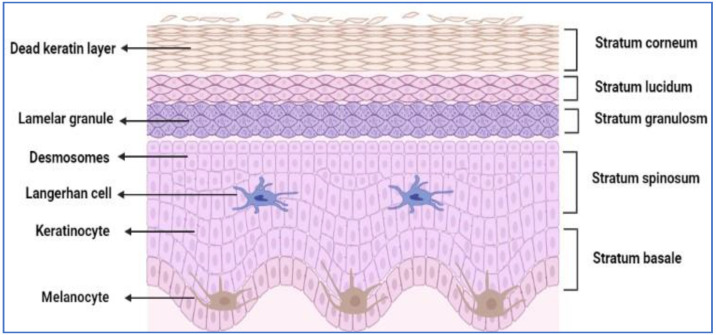
Schematic representation of the epidermis layer.

The epidermis consists of the stratum granulosum, stratum spinosum, stratum germinativum, and stratum corneum, the most remote sub-layer of the epidermis [3]. SC is approximately 15 µm thick, consists of layers of keratinised corneocytes and is segregated by an intercellular lipid domain [4]. The schematic representation of the epidermis is shown in Figure 2. The intercellular lipid domain consists of neutral lipids, ceramides, triglycerides, and free fatty acids. Other remaining components are phospholipids, glycosphingolipids, and cholesterol, which are important for the desquamation process. The corneocytes in the lipid–protein matrix are arranged in a brick wall formation. The corneocytes are the bricks, and the lipid–protein matrix is the mortar. The thick overlapping corneocytes are held together by corneodesmosomes and are entrenched in a complex mixture of intercellular lipid matrix. These contribute to the tightness and impermeability characteristics of the intact skin and thus form a major permeability barrier for hydrophilic molecules bearing a molecular mass greater than 200–350 Da [5].

### 2.2. Dermis

The dermis is a hydrophilic deposit with a thickness of 0.1–0.5 cm. The dermis comprises a network of elastin and collagen fibres entrenched in the mucopolysaccharide matrix that consists of blood vessels, lymphatic and nerve ending, pilosebaceous units, and sweat glands. The collagen fibres in the connective tissue provide support, whereas the elastic tissue gives flexibility. The dermis does not noticeably inhibit the transfer of substances (although it may be a significant hurdle for extremely hydrophobic drugs) [6]. Blood vessels in the dermis domain remove substances that pass through the epidermis layers, preserving the concentration gradient between the dermis and skin surface that impels penetration. In addition, sebaceous glands, sweat glands, and hair follicles also instigate the dermis region and create a “shunt” pathway favouring a few permeants.

### 2.3. Hypodermis

The hypodermis, or subcutaneous layer, is the innermost layer of the skin. It performs like an absorber to any shock drawn towards the body and facilitates insulation [7]. Hypodermis consists of different cells, including connective tissue, adipose tissue (fat cells), fibroblasts, blood vessels, and macrophages.

## 3. Permeation Process

**Figure 3:**
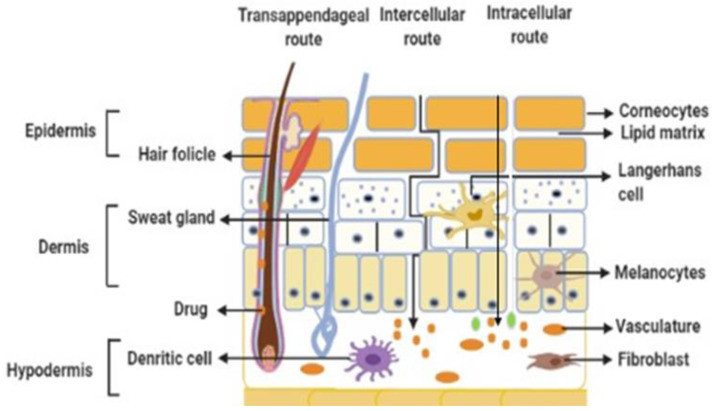
Schematic representation of the permeation process through the skin.

Drug molecules can cross the skin barrier and enter the systemic circulation by different routes. These routes include intracellular, intercellular and transfollicular routes, as shown in Figure 3. The intracellular route is a potential course of diffusion. However, diffusion’s main course emerged through the intercellular spaces in the investigational study. This is due to the presence of hydrated keratin, the protein cell envelope, covalent lipid monolayer, and free intercellular lipids across this pathway. Most drugs do not cross the SC through this pathway due to its numerous partitioning-diffusion stages through the hydrophilic and lipophilic domains. For example, highly lipophilic drugs will not be able to penetrate the hydrophilic domains when it follows the intracellular pathway.

In contrast, they will have the capability to traverse the lipophilic domains due to their likeliness nature. However, in the case of hydrophilic drugs, they are more likely to transverse across the SC through the defects in the lipids surrounding the corneocytes. The intercellular pathway continuously passes through the lipid matrix through the SC layer and is present around the corneocytes. The intercellular pathway does not change the penetration direction between the initial application and establishing a favourable steady state. Therefore, it is considered the most favourable pathway for small molecules [8]. The time required to establish a favourable steady state does not affect the absorption rate and the permeation process. The transfer of molecules through this course engaged in sequential diffusion and partitioning amid the alkyl chain length and the polar head groups of the intercellular lipids. The total surface area occupied by the sweat glands and hair follicles is approximately 0.1%; therefore, it is not considered a noteworthy alleyway for drug permeation [9]. Furthermore, before attaining a steady-state concentration, the transfollicular course might take part in the speedy diffusion of drugs in the initial hours. Nevertheless, the transport of drugs through this course may conceivably be imperative for high molecular weight drugs. Until now, various approaches have been employed for enhancing drug transport across the skin, including drug–vehicle interaction, vesicles and their analogues, modification of the horny layer, removal of the horny layer, and electrically-driven techniques. Drug–vehicle interaction is further classified into techniques such as drug and prodrug selection, ion pair, and eutectic mixtures. Vesicles and their analogues are subdivided into liposomes, ethosomes, niosomes, and transferosomes. Modification of the horny layer is achieved by employing various types of CPEs, including water, alcohol, terpenes, azone, sulfoxides, surfactants, phospholipids, and urea. On the other hand, the removal of the horny layer is achieved using microneedle techniques. Lastly, electrically-driven techniques include ultrasound, iontophoresis, electroporation, magnetophoresis, and photomechanical wave. Different approaches that are enumerated above are thoroughly discussed in the upcoming sections.

## 4. Marketed Transdermal Products 

A TDDS is a good replacement for conventional drug delivery approaches such as oral administration and injection. Nevertheless, the commercial application of a TDDS is restricted because only a few drugs can be effectively transported across the skin at the actual rate. However, several transdermal products increase drug transport across the viable skin. Transderm Scop^®^ was the first transdermal product marketed in 1979 and was used for treating motion sickness at sea for 3 days. Moreover, it was compared with oral administration, showing a better result in reducing the side effects. Succeeding the scopolamine transdermal product, a clonidine-containing transdermal patch, Catapress-TTS^®^, was approved in 1984 for the treatment of hypertension. Other transdermal products, such as Estraderm^®^ and Harbitrol^®^ and Duragesic^®^, were developed and marketed in 1986 and 1990, respectively. The development of transdermal products containing hormones started in 1991 until 2004, which includes oestradiol, testosterone, ethynyl estradiol, norelgestromin and levonorgestrel. This proposes that in the inception period, transdermal products were predominantly projected for the transport of hydrophobic drugs, which are composed of sterols [10].

Various categories of drugs were incorporated into a transdermal product from 2005 to 2013, including selegiline (Emser^®^), methylphenidate (Daytrana^®^), fentanyl (Ionsys^®^), diclofenac epolamine (Flector^®^), a combination of menthol/methylsalycylate (Salonpas^®^), and sumatriptan (Zecuity^®^). Lately, in 2019 and 2020, the US FDA has approved two transdermal products, Secuado^®^ (asenapine for schizophrenia) and Twirla^®^ (ethinyl estradiol and levonorgestrel), respectively [11]. 

Nanotechnology-based approaches have a great ability to produce effectiveness; however, the full potentials are theoretical and have yet to be explored. Presently, the major drawbacks of the molecular manufacturing outline have not yet been fully explored. Henceforth their pros and cons will continue to be the dominant focus of researchers. Numerous products of therapeutics based on self-regulation, ultra-adaptability, and nano-sized particles are being marketed. Some of them also have reached the clinical level. A topical liposomal formulation which consists of econazole as an active ingredient (an antifungal drug) has been approved in Switzerland, and VivaGel is another example of a dendrimer-based formulation and it was developed by Starpharma [12,13]. NB-00X is another topically applied product developed by NanoStat technology for herpes labialis. IDEA AG, a well-known biopharmaceutical company, publicized the start of the development of targeted therapeutics for the treatment of osteoarthritis related to the knee based on the novel transfer of some carriers (topically applied dosages of IDEA-033) and they are currently in phase III trials in Europe. However, several queries arose, which have continued to be unexplored and addressed. Safety concerns that affect health are important issues for the manufacturing of these particles. Lastly, it is expected that the use of nanotherapeutics is boundless, but the development of safety guidelines by the manufacturers must be strongly considered [14,15].

Several microneedle devices are already well-established marketed products (e.g., Dermaroller, Dermapen) with high customer acceptance, exclusively for cosmetic purposes; existing research emphasises shifting toward polymeric dissolving and biodegradable microneedles for the treatment of systemic disease. Currently, the goal of designing a microneedle array is not merely to increase the transdermal delivery of a particular drug but also to respond to the biological surroundings and attain a sustained release. Moreover, the additional emphasis of the microneedle is on transforming transdermal delivery approaches from bench to bedside. Undeniably, there are about 39 completed and 23 active National Institutes of Health (NIH) clinical trials relating to microneedle techniques [16]. A majority of the clinical trials presently employ solid non-dissolving microneedle systems, whereas in a few cases, dissolving microneedles had also been used. Even though a wide array of research has been carried out and has successfully established transdermal delivery efficacy using the microneedle technique, numerous challenges are yet to be addressed.

## 5. Drug–Vehicle Interaction

**Figure 4:**
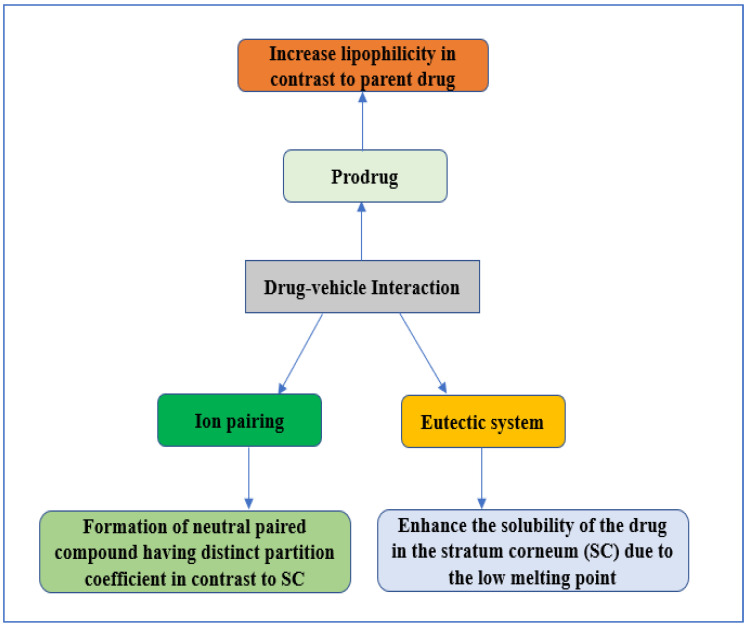
Permeation process through the skin via drug–vehicle interaction.

Drug–vehicle interaction is one of the earliest methods used to enhance skin absorption. In particular, drug–vehicle interaction is classified into four distinct methods: prodrug selection, eutectic system, ion pairing, and chemical potential [17]. The main intention of this strategy is to enhance skin permeability by modifying the SC without affecting the skin layers beyond the SC, and the permeation process through the skin via drug–vehicle interaction is shown in Figure 4. This section discusses prodrug, ion pairing, and the eutectic system as examples of drug–vehicle interaction.

In 1958, Adrian Albert first introduced the term “prodrug”. In context of drug delivery, prodrugs are one of the most beneficial formulations with altered physiochemical properties, which turn them pharmacologically inactive until they are metabolised inside the body to form their active drug moiety. Prodrug experiences biotransformation ahead of showing their therapeutic effect. The sole reason for manufacturing a prodrug for transdermal delivery is to develop a compound with a log *p* value of 1–3. The correlation between the log P value and lipophilicity is that as the value of log P decreases, lipophilicity eventually increases and vice versa. Since most synthesised prodrugs have a low log P value, they eventually possess high lipophilicity and higher transdermal flux than their parent moiety. The partition coefficient also has an essential role in defining the permeability across the skin. However, log P stands out as a more significant factor to relate to the skin permeation of a drug. Another important parameter affecting the prodrugs’ permeation is the diffusivity (D), which depends on the molecular weight. The diffusivity of similar prodrugs has to be related inversely to the third root of their molar mass to prevent any fluctuations in the flux due to a change in the diffusivity. Many studies have been carried out on transdermal prodrugs, including naproxen, stavudine, morphine, haloperidol, naltrexone, cycloserine, indomethacin, and bupropion [17,18,19,20,21,22,23,24].

Ionised drugs usually have undesired physicochemical properties that hinder the absorption of drugs across the SC, eventually making them suitable for the ion-pairing method. In an ion-pairing method, a neutral paired compound is formed by adding counter ion species with a distinct partition coefficient in contrast to the SC [25]. The subsequent transdermal application of the neutral paired compound initiates the release of the parent drug and finally enters the systemic circulation via absorption. The partition and diffusion mechanism through the SC releases the drug from the neutral paired compound before it alienates into a viable epidermis. The ion-pairing method is used for numerous drugs to enhance the transdermal permeability across the skin. Examples of drugs include nicotine, risedronate, berberine, bisoprolol, zaltoprofen, and escitalopram [26,27,28,29,30,31]. Another commonly used method for enhancing skin permeability is the eutectic system. A eutectic system comprises two components; when mixed, it exhibits a lower melting point than either. The improved permeation through the skin is due to the low melting point of the mixture, which is assumed to enhance the drug solubility in the SC. A study has been conducted using a eutectic system to increase the skin permeability of several drugs, including ibuprofen, propranolol, lidocaine, flurbiprofen, aceclofenac, tenoxicam, meloxicam, and risperidone [32,33,34,35,36].

## 6. Vesicles and Their Analogues

**Figure 5:**
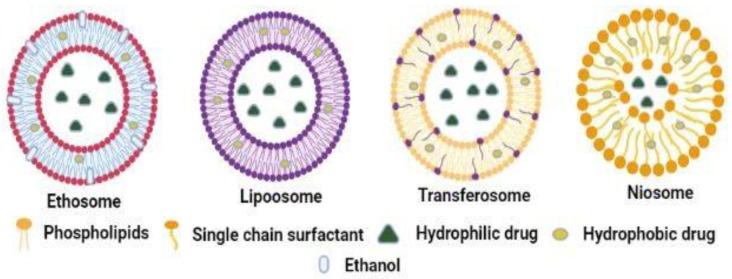
Structure of ethosome, liposome, transferosome, and niosome.

After drug–vehicle interaction, interest has increased in using vesicles and their analogues for the transdermal delivery of various drugs. By definition, nanovesicles are sphere-shaped bilayer vesicles made up of components such as lipids, alcohol, and surfactants [37]. Liposomes, ethosomes, transferosomes, niosomes, and phytosomes are nanovesicles used to enhance skin permeability. Various nanovesicle parameters, such as composition, size, surface charge, and deformability, affect drug diffusion across the skin. Numerous concepts are assumed for nanovesicle skin penetration, such as the adsorption effect, penetration through the transappendageal route, entire vesicle penetration, and interaction with SC lipids that results in limited fluidization. In particular, nanovesicles with a size greater than 600 nm are naturally unable to penetrate the skin layers; however, nanovesicles with a size smaller than 300 nm have the potential to enter the deeper epidermal region and dermal strata. Some researchers have delineated that drug penetration through the skin is affected by altering the surface charge of nanovesicles. The lipid in the SC comprises a high ratio of negatively charged lipids and makes the skin act as a negatively charged membrane [38]. It has been suggested that the existence of charges at the surface of nanovesicles may affect the transcutaneous diffusion of drugs. Moreover, nanovesicles with a negative charge on the surface usually produce a higher flux than their positively charged counterparts, enhancing the accumulation of the drug in the superficial skin strata [38]. The composition and deformability of nanovesicles vary, and their influence on drug diffusion across the skin is briefly discussed below. The different types of nanovesicles are described in this section, and the schematic of their structures is given in Figure 5.

Liposomes are composed of phospholipids and cholesterol and have one or more bilayer structures. Phospholipids are the key components of liposomes and are amphiphilic as they comprise both polar heads and non-polar tails. Owing to these features, liposomes can encapsulate both lipophilic and hydrophilic drugs [39]. A lipophilic drug is encapsulated between the lipid bilayer of the liposomes, whereas a hydrophilic drug is encapsulated in the liposome core. Liposomes disrupt the outer layer of the SC, in which the phospholipid components act as permeation enhancers that assist drug penetration into the skin. Liposomal-based transdermal drug delivery has been used for numerous drugs. Examples of drugs include diclofenac, ketoprofen, baicalein, vitamin C, amphotericin B, and azithromycin [40,41,42,43,44,45]. Ethosomes are the second type of nanovesicles employed to enhance transdermal drug delivery. Phospholipids and alcohol (20–40%), such as ethanol, are the main components of the ethosome. The main objectives behind using ethanol are to improve the flexibility of conventional liposomes and to act as a skin penetration enhancer for a drug. Due to improved biocompatibility and enhanced drug permeability, it is considered a superior technique than liposomes [46]. Numerous drugs, such as curcumin, valsartan, indomethacin, quercetin, mitoxantrone, econazole nitrate, apigenin, and green tea extract, are delivered through the skin membrane by using ethosomes as a carrier system [47,48,49,50,51,52,53,54].

Transdermal absorption of drugs can be improved by ethosomes, which are nanocarriers with exceptional deformability and drug-loading capacity. Ethanol within the ethosomes increases the membrane fluidity and permeability of the phospholipid, leading to drug leakage. To address this issue, a new phospholipid nanovesicle containing ethanol co-hybridized with hyaluronic acid (HA) was developed, and volatile oil medicines (eugenol and cinnamaldehyde [EUG/CAH]) were encapsulated for transdermal administration. The results suggested that in contrast with EUG/CAH-loaded ethosomes (ES), the stability and transdermal absorption of EUG/CAH-loaded HA-immobilized ethosomes (HA-ES) were significantly enhanced [55]. Transethosome, a modified version of ethosome, has gained much interest in transdermal drug delivery. It comprises phospholipids, surfactants, and a higher amount of ethanol which act as permeation enhancers. This has been explored for several drugs, such as piroxicam, agomelatine, paeonol, fisetin, and epigallocatechin gallate-containing extract [56,57,58,59,60]. Transferosomes are ultra-deformable liposomes consisting of a phospholipid and a rim activator commonly known as a surfactant with a single chain.

The ultra-deformable structure of transferosomes is attributed to the rim activator, which disturbs the lipid bilayer of transferosomes, providing additional flexibility compared to a normal liposomes [61]. The ultra-deformable structures of transferosomes allow deeper penetration into the skin layer, employing elastic transport. Moreover, the hydration of the skin and the osmotic process also enhance the penetration of transferosomes [62]. Several drugs, such as raloxifene, cilnidipine, pentoxifylline, diflunisal, and minoxidil, are formulated and successfully delivered using transferosomes [63,64,65,66,67]. Niosomes are surfactant-based nanovesicles used to enhance transdermal drug delivery. They are composed of a surfactant, specifically non-ionic, and cholesterol. The non-ionic surfactants form the bilayer structure, while cholesterol imparts rigidness to the structure [68]. Since non-ionic surfactants possess both the hydrophilic head and the hydrophobic tail, they can encapsulate polar and non-polar compounds. Niosomes have been explored for the transdermal delivery of numerous drugs, such as sumatriptan, resveratrol, salidroside, atenolol, and sulfadiazine [69,70,71,72,73]. Recently newer types of niosomes, such as cholesterol or phospholipid-free niosomes, have gained enormous attention in tropical and transdermal drug delivery. A study was conducted by designing multilamellar niosomes (MLNs), which are cholesterol and phospholipid free, with the help of glyceryl monooleate (GMO) and poloxamer 407 (F127), and they were evaluated for their capacity for transdermal drug delivery. The mean size of the optimized MLNs was 97.88 ± 63.25 nm with 82.68% ± 2.14% encapsulation efficiency. The skin deposition study reveals that MLN shows lower transdermal flux than the tincture, but higher skin deposition of aconitine was achieved in the MLN group (*p* < 0.05). In addition, both rhodamine B-and coumarin 6-labeled MLNs were found to permeate into the deep skin through the hair follicles and could be internalized by fibroblasts. Moreover, it was also confirmed that MLNs were inferior to the hydrophobic PLGA nanoparticles (diameter: 637.87 ± 22.77 nm), which mainly accumulated in superficial hair follicles. The hair follicles pathway significantly improves drug permeation [74].

Microemulsions (MEs) are the isotropic, transparent, heterogeneous system of two immiscible liquids (oil phase and aqueous phase) and an emulsifier commonly known as a surfactant (co-emulsifier/co-surfactant). It is a thermodynamically stable but kinetically unstable system and typically possesses a nanodroplet in a size range of <100 nm or less [75]. Generally, MEs are classified into three types, i.e., O/W (where the oil phase is dispersed in the aqueous phase), W/O (where the aqueous phase is dispersed in the oil phase), and bicontinuous emulsion, where micro domains of oil and water phases are inter-dispersed within the system. MEs are also classified based on their surface charge, i.e., neutral, anionic, and cationic [76]. Another important feature of the microemulsion is the spontaneous formation process, which does not require significant energy and is different from nanoemulsions usually prepared with ultrasound or high-shear homogenization [77]. Emulsifier selection is based on their solubility in oil and aqueous phases, HLB value, toxicity profile, etc. The oils play a vital role in ME formulation and are responsible for solubilizing active drugs with inherent low solubility. The amount of oil may vary from 2 to 20% w/w based on the administration site. These excipients possess excellent stabilizing properties, promote alterations in the SC layer, and facilitate penetration of active drugs across the lipid-rich SC layer. These are the classic examples of chemical penetration enhancers (CPEs) that can disrupt the lipid-rich SC layer and increase the solubility of the active drugs inside the system. In addition, the presence of surfactants over the droplet surfaces offers excellent skin permeability and improves the retention time of therapeutically active drugs. ME have been employed for the transdermal delivery of many drugs, including indirubin, rasagiline, insulin, levamisole, baclofen, astilbin, and nifedipine [78,79,80,81,82,83,84]. 

Mueller et al. were the first to introduce and explore the potential of solid lipid nanoparticles (SLNs) in drug delivery in the mid-1990s [85]. SLNs are colloidal particulate systems with a size range between 100 and 400 nm and are composed of lipid matrices that are biocompatible and biodegradable. In addition, they include another crucial component, which is an emulsifier. Solid lipids commonly used to prepare SLNs are stearic acid, palmitic acid, tripalmitin, trimyristin, tristearin, trilaurin, and tricaprin. In addition, advancement in lipid chemistry has forged a way to develop specialized solid lipids that include Compritol^®^ 888 ATO, Percirol^®^ ATO 5, Softisan^®^ 100 and 142, Witepsol H 35, Witepsol W 35, Witepsol E 85, Witepsol S 55, glyceryl monostearate, and glyceryl palmitostearate. To understand the molecular perspective of SLNs, it is essential to understand the physicochemical properties of these lipids. Among these physicochemical properties, the melting point and the polymorphic form of the lipids play a great role in the formation of SLNs. Emulsifiers used to formulate SLNs are soyabean lecithin, egg lecithin, phosphatidylcholine, poloxamer 188, poloxamer 407, Tween 20, Tween 80, taurocholic acid sodium salt, and dioctyl sodium sulfosuccinate. Due to the colloidal nature, they possess the advantages offered by other colloidal drug carrier systems such as nanoemulsion and liposomes. Several advantages make SLNs superior to the aforementioned colloidal drug carrier system, including the particulate nature of SLNs, their ability to encapsulate both types of drugs (hydrophilic and lipophilic), their ability to release the incorporated drug in a controlled manner, their ability to avoid degradation, their ability to immobilize drug in the solid matrix, and the simplicity in development and scale-up. SLNs have been explored for the transdermal delivery of numerous drugs, such as hydroxyzine HCL, loteprednol etabonate, curcumin, lovastatin, flurbiprofen, pranoprofen, and curcumin [86,87,88,89,90,91,92].

## 7. Chemical Permeation Enhancers (CPEs)

**Figure 6:**
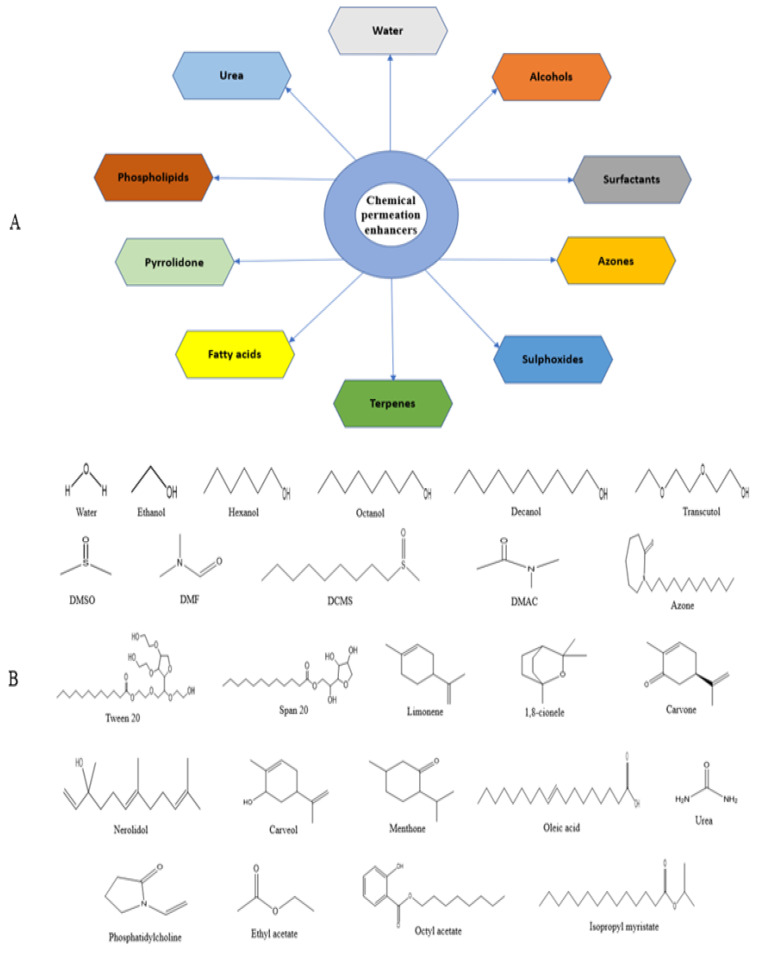
(**A**) Various types of chemical permeation enhancers used in TDDSs and (**B**) 2D-chemical structures.

In TDDSs, CPEs act as SC modifiers without causing any damage to the skin and lead to enhanced drug permeation. Morrow et al. indicated that using chemical permeation enhancers is one of the classic approaches for altering the SC. Various chemical permeation enhancers are shown in Figure 6 and are discussed in terms of their mechanism of action and toxicity profile.

### 7.1. Water

Employing water as a penetration enhancer is a classical approach to advancing transdermal drug delivery. The human SC consists of 15–20% water and is found in two states. The first one is the ‘bound water’, which constitutes 25–30% of the water present in the SC; the second one is the ‘residual water’ or ‘free water’ present inside the membrane that works as a solvent inside the membrane specifically for polar permeates [93]. The SC also restrains other components, such as amino acids and corneocytes, with functional groups, such as alcohol and a carboxylic acid, which bind with water molecules, preserve water inside the SC, and facilitate tissue elasticity. The mechanism by which hydration conditions enhance the delivery of lipophilic drugs is described by the fact that water molecules intensify fluidity in the cholesterol-stiffened domain and improve interactions between head groups. However, Bouwstra et al. revealed that the water did not induce SC modification [94]. This leads to the question: ‘‘What causes the SC layer modification?” One may anticipate that the corneocytes in the SC would imbibe water and swell up, which would cause modification of the SC layer.

Nevertheless, the results from electron microscopy of the completely hydrated SC illustrate that the lipid bilayers enclose puddles of water molecules with a sac-like configuration with no coarse deformation to the lipid bilayer packing [95]. There is also a belief that a pathway in the SC is aqueous. These pathways are formed under extensive hydration, and the creation of such pathways might distinctly increase drug permeation. The effects of water on transdermal permeation may vary from species to species depending upon the response. For example, Bond and Barry et al. found out that the shaved skin of a mouse was not suitable as a model when probing the hydration effects as the permeability of the shaved skin increases 50-fold, which is in contrast to the outcomes attained from human skin [96].

### 7.2. Alcohols

Alcohols are the most common CPEs exploited in transdermal drug delivery and are frequently used as co-solvents and water. Alcohols are categorised as short-chain solvents; ethanol can remove the SC lipids when applied at optimum concentrations for long periods [97]. Ethanol’s impact on estradiol’s skin permeation was investigated using the human skin sandwich flap model. An in vivo flux in ethanol or ethanol solutions across viable human skin was increased with a saturated solution of estradiol [98]. The absorption of ethanol and water into SC was investigated. The key mechanism for increasing skin permeation appears to be the miscibility of ethanol with water to interact with keratins. A symmetrical result was evident for other drugs, such as salicylate and ibuprofen [99,100]. However, due to the fast evaporation and limited application period, ethanol’s role in enhancing the drug’s permeation is restricted, for example, in the Durogesic^TM^ reservoir patch [101]. In 1991, Janssen’s Duragesic reservoir fentanyl transdermal system was approved clinically for treating chronic and cancer therapy-related pain. Duragesic consists of a rate limiting membrane intended to provide a continuous systemic delivery of fentanyl, which is the innovation and, subsequently, the reference product. Duragesic is available for clinical use in five strengths-12, 25, 50, 75, and 100 mg/h, each intended to offer 72 h of dosing in a single application. The quantity of fentanyl released per hour from each system is proportional to the surface area of the patch (25 mg/h per 10 cm^2^). The composition per unit area is equal for all dosage strengths. The Duragesic patches contain 0.1 mL of alcohol per 10 cm^2^ as a penetration enhancer [102]. Long-chain alcohols also have penetration-enhancing activity and are usually used at a concentration between 1% and 10%.

Structure-activity relationships for long-chain alcohols have indicated that branch alkanols possessed lower activities, whereas 1-butanol was the most efficient enhancer for levonorgestrel traversing rat skin [103]. Another study suggests that the permeation of melatonin depends on the carbon chain length and its number of double bonds [104]. The effects of hexanol, octanol, and decanol were investigated using FT-IR spectroscopy and tape stripping. The results suggested a shift in solvent uptake due to the C-H stretching frequency. Lipid disorder was generated by all the vehicles relative to the concentration of vehicles in the skin [105]. Transcutol (TC) is a hydrophilic CPE possessing comparable solubility parameters with the skin. It is generally employed in transdermal and topical formulations due to its capability to increase permeation. The main mechanism of this solvent is to escalate the partition of the drug into the skin, which may be due to the solubility parameter of TC that is close to the skin. TC infiltrates and engages in the skin membrane in maximum quantities in contrast to other hydrophilic CPEs; as a result, a moderate quantity of the drug permeates through the skin. In addition, it illustrated the ‘pull’ effect assisting superior absorption of drug molecules [106]. Recently, penetration enhancer-containing vesicles (PEVs) as carriers for enhanced transdermal drug delivery have gained much attention. A study was conducted by developing a Transcutol-containing PEVs as carriers for diclofenac in the form of either acid or sodium salt. The prepared PEVs were characterized by their size, entrapment efficiency, and stability.

Moreover, an ex vivo skin penetration study was also executed for conventional liposomes and a commercial gel as controls. The all-skin permeation experiments showed an improved diclofenac (both acid and sodium salt) delivery to and through the skin when PEVs were used (especially in comparison with the commercial gel), thus suggesting intact PEVs’ penetration through the pig skin. So, these studies confirm the superiority of the PEVs in enhancing ex vivo drug transport of both diclofenac forms [107]. Another study was conducted to determine the flux of sodium naproxen incorporated in Pluronic F-127 (PF-127) gels comprising two penetration enhancers, Azone and Transcutol, through human skin in vivo. Results of the study suggested that the combination of Azone and Transcutol in PF127 gels increases the penetration of sodium naproxen with enhancement ratios of up to two-fold compared with the formulation containing only Transcutol. The finding was confirmed by TEWL and ATR-FTIR spectroscopy, suggesting a synergic action for Azone and Transcutol [108]. The study was conducted for the transdermal drug delivery of thymoquinone (TQ) and to evaluate the effect of ethanol and propylene glycol as donor solvent systems and various compositions of receptor solvents. In addition, the effects of penetration enhancers were studied using human cadaver skin. The permeation of saturated solutions of TQ was investigated with 5% *v*/*v* of each of the following permeation enhancers: Azone (laurocapram), Transcutol^®^ P (Tc), oleic acid, ethanol, Tween 80, and N-methyl-pyrrolidone (NMP). The results revealed that Azone, oleic acid, and Tc could deliver adequate TQ flux. The authors also indicated that these penetration enhancers were proficient enough to produce TQ reservoirs which may be beneficial to release the drug at a sustained rate [109]. 

### 7.3. Sulfoxides

Dimethylsulphoxide (DMSO) is the most commonly used penetration enhancer among sulfoxides. It is a potent aprotic solvent that is pale, unscented, and hygroscopic. DMSO produces its enhancement activity by modifying the intercellular keratin from α helical to β sheet [110]. Radio-labelled and non-labelled DMSO measured its penetration capability into the human skin. The results suggest that 15–30% of topically applied DMSO penetrates human skin in vitro within 2 h, while in vivo results indicated that DMSO was metabolised in the body. In vitro quantification of 14C-labelled fluocinolone acetonide, triamcinolone acetonide, and hydrocortisone using DMSO and 95% alcohol as vehicles indicates that DMSO possessed higher penetration power [111]. An analogous study was conducted using radio-labelled hydrocortisone and testosterone, and DMSO unequivocally increased the penetration of both drugs [112]. The in vitro penetration of fluocinonide across the human skin was studied in the presence of DMSO. The thermodynamic activity of the drug’s penetration through the skin was increased by DMSO [113]. A comparative study was conducted using ethanol, DMF, and DMSO as penetration enhancers for bepridil. It has been noted that DMSO performed as a true penetration enhancer at a concentration of 50% [114]. The efficacy of DMSO was conducted by employing molecular simulations, and the outcome suggests that the DMSO concentration must be high to be efficacious [115]. Nevertheless, DMSO at high concentrations can cause erythema, scaling, contact urticaria, stinging and burning feelings and produce a malodorous metabolite in the breath.

As DMSO produces undesirable side effects, researchers have examined several chemicals related to DMSO. Dimethylformamide (DMF) and dimethylacetamide (DMAC) are other aprotic solvents with structures analogous to DMSO. An in vitro study was conducted on DMF-treated human skin to find out the possible mechanism by which DMF enhances the flux of caffeine. It has been concluded that DMF causes irreversible membrane damage. New structural analogues, such as decyl methyl sulphoxide (DCMS), have been primed. DCMS is concentration-dependent and acts reversibly on human skin. The literature on DCMS reveals that it is a strong penetration enhancer for hydrophilic permeants and less effective for hydrophobic permeants [116].

### 7.4. Azone

Azone was prepared as a transdermal penetration enhancer and was patented in as early as 1976 [117]. Chemically, Azone is considered a fusion of a cyclic amide with alkyl sulfoxide that does not contain an aprotic sulfoxide group producing very low irritancy. In addition, it is extremely hydrophobic but soluble in a majority of organic solvents. The effectiveness of Azone emerges to be concentration dependent, with it being employed in a range of 1–3%. Azone interacts with the SC lipid, which may exist as a separate domain within the SC lipid forming a ‘soup spoon’ conformation. Electron diffraction studies suggest the existence of an Azone as a separate phase within the SC lipids [118]. Franz diffusion technique and ATR-FTIR spectroscopy were employed to analyse the penetration mechanism of cyanophenol, and the flux of cyanophenol was increased by Azone by reducing the diffusional resistance of SC and producing a more fluid environment [119].

Furthermore, the effect of Azone on lipids and water mixtures was investigated using wide- and small-angle X-ray diffraction techniques. The results establish three phases at room temperature, one gel and two crystalline phases [120]. The penetration of naproxen across the rabbit ear skin and human skin was determined, and it was found that Azone increases the penetration by up to 4-fold [121]. Hadgraft et al. proposed that Azone can form ion pairs with anionic drugs, promoting permeation [122]. The study was conducted to optimise the permeation efficacy of Azone in combination with salicylic acid using optical coherence tomography (OCT) and diffuse reflectance spectroscopy (DRS). The study’s outcomes suggested that azone, in combination with salicylic acid, produces a synergistic effect based on the penetration of light and OCT imaging depth [123]. Celecoxib was delivered topically using Azone as a penetration enhancer. The results have shown higher retention of celecoxib in the epidermis and dermis levels, illustrating a localised celecoxib effect [124]. The effects of numerous penetration enhancers on the transdermal delivery of thymoquinone were studied using human cadaver skin in Franz diffusion cells. The results suggested that Azone and other penetration enhancers could provide sufficient flux and create thymoquine reservoirs in the skin [109].

### 7.5. Surfactants

Anionic surfactants bear a negative charge in their hydrophilic part, and examples of anionic surfactants include soaps, sodium lauryl sulphate (SLS), dioctyl sodium sulphosuccinate, and phosphate esters. Anionic surfactants bind to the proteins in the epidermal region, increasing the anionic sites in the membrane and enhancing the hydration intensity. A DSC study was conducted to determine the effect of SLS on promoting the skin’s hydration level, and the result signifies that the water content in the tissue increases due to SLS [125]. Another study observed that the carbon chain length of an anionic surfactant also affects the skin’s hydration [126]. However, as the carbon chain length increases, the irritation also increases, and the highest response was observed for the C_12_ analogue of SLS. A comparative study was conducted between SLS and other surfactants on in vitro skin permeation of ketotifen. SLS showed the most prominent effects and drastically enhanced the permeation at concentrations over 1 mM [127]. A similar study was conducted using lorazepam as a model drug, and SLS at 5% *w*/*w* showed the highest flux of lorazepam [128]. With the utilisation of low-frequency ultrasound combined with an anionic surfactant, the penetration of polar chemicals, such as mannitol, was increased [129]. In addition, anionic surfactants produce a reversible action since the skin tissues revert to their standard form upon surfactant removal [130].

Cationic surfactants carry a positive charge on the hydrophilic head group with bulky lipophilic hydrocarbon groups and are often quaternary ammonium compounds. CTAB and BKC are the commonly known cationic surfactants used in transdermal formulations to enhance the permeation of various drugs, including diazepam, haloperidol, and methyl nicotinate [131,132,133]. Cationic surfactants exert their permeation effect by swelling the SC and interacting with intercellular keratin. However, these compounds were not assessed in vivo for penetration enhancement due to their serious side effect.

Non-ionic surfactants have a polar head group with a hydrophilic cluster of non-dissociable types. This class of non-ionic surfactants enhances permeation by interacting with the SC lipids and increasing the membrane’s fluidity. Non-ionic surfactants generate fewer irritating sensations and are generally considered safer than ionic surfactants. An in vitro study was conducted using human skin to support the above assumption. It was revealed that only ~0.5% of the applied dose traversed human skin [134]. Among the non-ionic surfactants, Tween and Brij series are the most commonly employed in permeation studies. An investigation was carried out to determine the effect of Brij 36T on the induction time of erythema by nicotinates when delivered transdermally. The results indicated that Brij 36T destructure the SC lipids and increases permeability [114]. However, in another study, applying 10% Tween 85 in petrolatum increased the water loss and enhanced the epidermal permeability [135]. 

In the last few decades, an enormous effort has been made to develop biosurfactants naturally processed by microorganisms when grown on water-miscible or oily substrates [136]. Numerous raw materials, predominantly carbohydrates, triglycerides, and organic acids, act as starting materials in biosurfactant synthesis. Triglycerides/sterols contribute to the hydrophobic part whereas sugars/amino acids contribute to the hydrophilic part of these surfactants [137]. Bio-based surfactants can decrease surface and interfacial tensions by using similar mechanisms as chemical surfactants. In addition, they possess numerous advantages compared to synthetic surfactants, including biodegradability, lower toxicity, improved surface and interfacial activity, higher selectivity and, hence, better safety and biocompatibility [137]. Glycolipids and lipopeptides are the best-studied microbial surfactants. Examples of glycolipids include rhamnolipids produced by *Pseudomonas aeruginosa*, trehalolipids produced by *Rhodococcus erythopolis*, sophorolipids produced by *Candida bombicola,* and mannosylerythritol lipids (MEL) produced by *Pseudozyma* yeasts, which contain mono- or disaccharides, combined with long-chain aliphatic acids or hydroxyaliphatic acids. Among the lipopeptides, examples comprise surfactin, iturin and fengicyn cyclic lipopeptides produced by *Bacillus* species as antibiotic molecules. Biosurfactant has been used in transdermal drug delivery as a permeation enhancer to increase the transport of various drugs across the skin, and examples include insulin, hydrocortisone, acyclovir, oestradiol, lactoferrin, and lignans [138,139,140,141,142,143,144].

### 7.6. Terpenes

Terpenes are a class of natural compounds regarded as safer than synthetic CPEs. Moreover, terpenes such as 1,8-cineole, menthol, and menthone are included under the Generally Recognised as Safe (GRAS) list. Terpenes can increase the permeation of hydrophobic and hydrophilic drugs, even at low concentrations, by affecting the SC lipids, specifically the intercellular lipids or the hydrogen bond connection in the SC lipid bilayer domain [145]. Terpenes commonly employed as penetration enhancers are 1,8-cineole, limonene, D-limonene, carveol, carvone, pulegone, nerolidol, L-menthol, and menthone. 1,8-cineole is the principal terpene element in eucalyptus oil and has been evaluated as a permeation enhancer for 5-fluorouracil and estradiol in human skin [146]. The effects of limonene and D-limonene were assessed for indomethacin and steroids using traverse rat skin. The result showed that limonene was more effective in increasing the drug flux [147,148]. A combination study was conducted for several terpenes such as carveol, carvone, and pulegone using propylene glycol as a vehicle, and the results have revealed that terpenes not as a whole increase the drug flux up to 4-fold rather than due to the synergistic effect of propylene glycol and terpenes [149]. Large terpene molecules, known as sesquiterpenes, have been evaluated as permeation enhancers for various drugs. One example of sesquiterpenes is nerolidol, which has been used to enhance the permeability of 5-fluorouracil up to 20-fold through human skin in vitro [94]. A comparison study was conducted on the cytotoxic effect of nerolidol and various monoterpenes in erythrocyte and fibroblast cells. The results have indicated that nerolidol showed a significant effect compared to monoterpenes [150]. L-menthol, a levo isomer of menthol, has been used to improve the in vitro permeation of imipramine hydrochloride across rat skin [151]. The exhaustive research conducted in the past makes it perceptible that the smaller terpenes tend to be more dynamic permeation enhancers than the larger terpenes. Additionally, the non-polar group containing terpenes provides improved permeation for lipophilic drugs; conversely, the polar group-containing terpenes improve permeation for hydrophilic drugs.

### 7.7. Pyrrolidone

Pyrrolidone is an organic compound consisting of a 5-membered lactam ring, and it has been used as a permeation enhancer for both hydrophobic and hydrophilic drugs in human skin. N-methyl-2-pyrrolidone (NMP) and 2-pyrrolidone (2P) are the most commonly explored pyrrolidones as penetration enhancers. An investigation was conducted using DSC on these two permeation enhancers (NMP and 2P); the results have indicated that these molecules increase lipid fluidity by interacting with the SC lipids [152]. Trommer et al. indicated that relatively hydrophilic pyrrolidones interact with the polar region of the SC. Meanwhile, hydrophobic pyrrolidones interact with the non-polar region of the SC [146]. Various penetration enhancers were screened for their activity depending on the skin’s electrical resistance changes. The results indicated that pyrrolidone enhances the penetration of melatonin in 48 h [153]. A virtual screening algorithm was built for generating impending CPEs. The results specify that only 1-dodecyl-2-pyrrolidinone and menthone provide adequate drug penetration with a low toxicity profile [154]. Though NMP presents a substantial increase in the penetration of various drugs, the use of these molecules was restricted since they were found to cause swelling, erythema, skin irritation, etc.

### 7.8. Fatty Acids

Oleic acid is an octadec-9-enoic acid with a double bond at C-9 with Z conformation. Owing to its desirable properties, it is used as a permeation enhancer in topical and transdermal formulations. Oleic acid produces activity by escalating fluidisation and skin diffusivity [155]. Oleic acid at higher concentrations presents as a separate phase within lipid bilayers. Subsequently, it can induce a discrete lipid domain within the lipid bilayer [156,157]. The creation of such phases would offer permeability flaws inside the lipid bilayer; consequently, it assists the permeation of hydrophilic drugs through the membrane and most likely results from the conformation of the double bond. ATR-FTIR measurements later confirmed this hypothesis on human volunteers treated with per-deuterated oleic acid [158]. Various model drugs have been investigated using oleic acid as penetration enhancers. A considerable effect on the SC has been observed in the replica membrane with a higher ratio of phytosphingosine-based ceramides [159]. Furthermore, a study was carried out on the permeation of oleic acid in rat skin using Raman spectroscopy. The results have indicated a time-dependent enhancement of oleic acid flux correlated with lipid peak changes [160]. Despite the benefit of oleic acid, substantial literature reports have stated that unsaturated fatty acids produce undesirable dermal side effects. Certain side effects can be overcome by reducing the acidic nature of the unsaturated fatty acid, which can be achieved through structural modification of the carboxylic terminal.

### 7.9. Phospholipids

Phospholipids (PL) are lipids consisting of a hydrophilic head and two hydrophobic tails coupled by an alcohol deposit. Phospholipids have been extensively employed to formulate nanoformulations in a vesicular structure, such as a liposome and ethosome, for topical and transdermal drugs intended for increased bioavailability, reduced toxicity, and increased flux across skin membranes. Phospholipids can blend with SC lipids by introducing them into the SC as vesicles. Nevertheless, few researchers have employed a non-vesicular form as a permeation enhancer. For instance, 1% phosphatidylcholine was used to increase the flux of theophylline across hairless mouse skin [161]. Correspondingly, 1% phosphatidylcholine in propylene glycol has been utilised to enhance indomethacin flux through rat skin. Other than phosphatidylcholine, phospholipids such as soybean phospholipid enhanced the permeation of diclofenac through the rat skin [162]. Considering the physicochemical properties and configuration of phospholipids, it is contemplated to interact openly with the SC lipids and present as a lipid region puddle. Meanwhile, no evidence is available about the interaction of phospholipids openly with SC lipids. 

### 7.10. Urea

Urea is an organic compound comprising two amines (‒NH_2_) groups attached to a carbonyl (C=O) functional group. It is pallid, scentless in nature, and possesses a very high solubility in water. Urea-containing topical and transdermal formulations promote skin rehydration because of their hydrotropic nature and have been used to manage psoriasis, xerosis, ichthyosis, and other hyper-keratotic skin diseases. Urea produces its penetration-enhancing activity, presumably by increasing the SC water content and keratolytic activity. Water in oil emulsion was formulated by incorporating urea, indicating significant SC hydration. Researchers have a keen interest in synthesising a more potent analogue of urea, which will impact transdermal research. Wong et al. synthesised cyclic urea analogues and established that their activities were similar to Azone in increasing indomethacin flux across hairless mouse skin [163].

## 8. Lipid Synthesis Inhibitors (LSIs)

**Figure 7:**
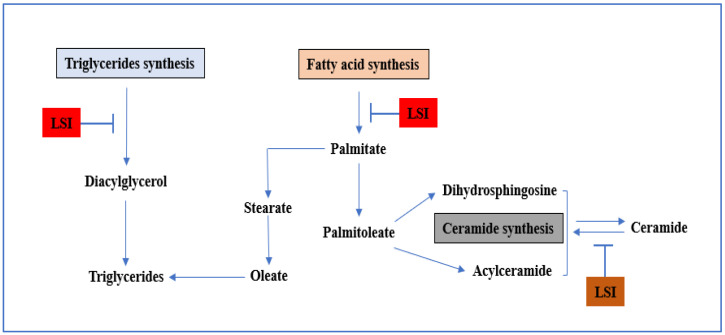
Mechanism of action of LSIs.

The SC lipids are mostly cholesterol, ceramides, fatty acids, and glycerol, which are the main reason behind the transdermal barrier function. A decline in the production of any of these critical lipid species compromises the barrier function, and the schematic representation of the mechanism of action of LSIs is shown in Figure 7. Considering the above consequences, it was hypothesised that transdermal drug permeability could be improved by interrupting or inhibiting skin lipid metabolism. A study was conducted using LSIs, such as 5-(tetradecyloxy)-2-furancarboxylic acid (TOFA), fluvastatin (FLU), and cholesterol sulfate (CS) for enhancing the transdermal delivery of lidocaine or caffeine and it was concluded that lipid synthesis inhibitor augments the transdermal delivery of the drug by altering both the barrier haemostasis or thermodynamic property of the skin [164]. Babita et al. performed a series of studies using LSIs, such as beta-chloralanine or atorvastatin, which superficially target the sphingosine (a precursor of ceramides), cholesterol cerulenin (an inhibitor of fatty acid synthase enzyme), and atorvastatin. In brief, all LSIs were used to enhance the transdermal delivery of levodopa by targeting various essential components in lipid synthesis [165,166,167]. Gupta et al. used beta-chloroalanine (beta-CA), a selective inhibitor of serine palmitoyl transferase, in combination with ethanol to increase the transdermal flux of 5-fluorouracil across rat skin [168]. Li et al. employed trypsin, a proteolytic enzyme, as a biochemical enhancer to increase insulin transdermal delivery [169]. Li et al. used trypsin to improve penetration via hair follicular delivery and the intercellular pathway. The ATR-FTIR study suggests that trypsin distorted the SC lipid structure, disturbing skin barrier properties and enhancing its effect [170].

## 9. Cell-Penetrating Peptides (CPPs)

CPPs are positively charged small peptides with 5–30 amino acids sequences that can penetrate biological membranes [171]. CPPs have attracted formulation scientists because of their high transduction efficiency and low cytotoxicity and are considered as a promising approach for transdermal delivery. The drug molecules intended to be delivered across the skin can be conjugated in covalent and non-covalent binding. A systemically efficient drug such as cyclosporine A (CsA) is inefficient topically due to its inherent poor penetration into the skin. To overcome this problem, Rothbard et al. conjugated a heptamer of arginine to CsA. The results have shown that unconjugated CsA fails to penetrate the skin, whereas conjugated CsA efficiently penetrates the skin and produces its therapeutic effect [172]. Kim et al. put forward a hypothesis stating that magainin, a small peptide recognised for its ability to form pores in bacterial cell membranes, can augment skin permeability by disrupting the lipid structure of SC. They conducted a skin permeation study to prove this hypothesis and concluded that magainin combined with N-lauryl sarcosine synergistically enhanced skin permeability 47-fold. In addition, magainin, in the presence of NLS-ethanol, extensively penetrates the SC and disrupts the SC lipid structure [173]. Interferon-gamma (IFN-gamma) possesses many therapeutic benefits, but one of the major drawbacks is the evidence of significant side effects when delivered systematically. Therefore, to reduce these side effects, Jung et al. fused a Pen (penetratin) peptide with IFN-gamma forming Pen-IFN-gamma. The outcome indicates that transdermal delivery utilising a Pen peptide may be a good approach to replenishing IFN-gamma in various disorders associated with this cytokine [174]. siRNAs are impending therapeutics for various skin diseases, and they are delivered through the skin using a peptide such as skin-penetrating and cell-entering (SPACE) peptide. In vitro studies specify that the SPACE peptide was capable of penetrating the SC [175]. A skin permeation study was conducted by fusing a novel cell-penetrating peptide (IMT-P8) with a green fluorescent protein (GFP) and pro-apoptotic peptide (KLA), forming two different domains. IMT-P8 was found to transport GFP and KLA across the mouse skin after topical application [176].

## 10. Ionic Liquids (ILs)

**Figure 8:**
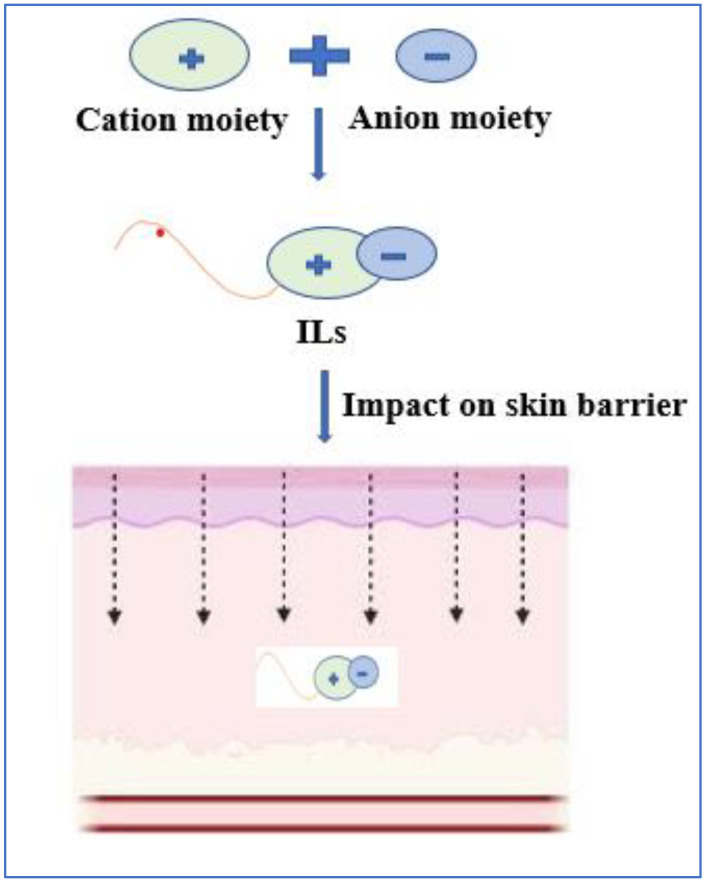
Formation of ILs and the effect on the skin barrier.

ILs are a group of chemical compounds with low vapour pressure, low melting point, high solubility, high thermal stability, and other tailor-made properties. ILs are made up of two components: cation and anion moieties. The diagrammatic representation of the synthesis of ILs and their effect on the skin barrier is shown in Figure 8. This amalgamation reduces the crystalline nature of the system, permitting ILs to be in liquid form at such low temperatures. ILs are extensively explored as chemical permeation enhancers in transdermal drug delivery [177]. ILs enhance the paracellular and transcellular, bypassing the barrier properties of SC [178]. The mechanism includes disruption of cellular integrity, fluidisation, creation of diffusional pathways, and extraction of lipid components in the SC [179]. The skin permeation of several drugs, such as donepezil, ibuprofen, acyclovir, hydrocortisone, and 5-fluorouracil was increased by using ILs as permeation enhancers, an integral part of the delivery system [180,181,182,183]. In addition, they have been used for certain biological drugs, including insulin, hyaluronic acid, framework nucleic acid, and siRNA [184,185,186,187].

## 11. Selection of CPEs 

CPEs have been used continously for decades to overcome the skin’s barrier properties and they have shown remarkable results in increasing numerous drugs’ availability, which is enumerated in Table 1. This extensive literature assessment shows that whilst selecting ideal CPEs for a particular drug, it is mandatory to consider their physicochemical properties and pharmacological and toxicological effects. The inherent physicochemical properties of penetration enhancers must be matched with the drug to attain an efficient permeation enhancement. CPEs produce their enhancing activity principally by diffusion, partition, and solubility. Modifying the intercellular lipid domains leads to fluidisation and reduces the barrier resistance of the lipid bilayers; furthermore, it intercedes the diffusion phenomenon across the skin. A similar effect was observed in the case of enhancers such as oleic acid and azone, which are proposed to subsist as an isolated segment in the intercellular lipid domains. The partition of the drug from the lipid bilayers of the SC is enhanced by creating an encouraging environment for solubility. These can be achieved by altering the solvent nature of the SC or introducing co-solvents into the tissue, raising the concentration of the permeants present inside the skin. These are mediated by the solvents that are absorbed into the skin well. Miscellaneous mechanistic intervention may include the disruption of desmosomes, which facilitate structural organisation among the corneocytes and change in the thermodynamic motion of the vehicle, as well as aid in the solubilisation of the drug in the donor compartment.

## 12. Microneedles

**Figure 9:**
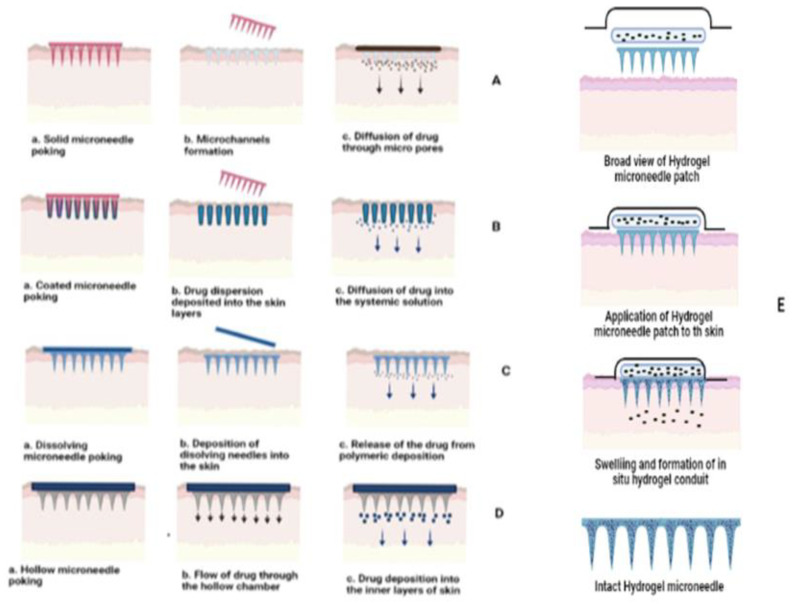
Microneedle approaches: (**A**) solid microneedles, (**B**) coated microneedles, (**C**) dissolving microneedles, (**D**) hollow microneedles, and (**E**) hydrogel microneedles.

Microneedles are a novel approach to delivering drugs by improving their percutaneous absorption. This dermal delivery system comprises micron-sized needles ranging from 1 to 100 microns in length organised above a transdermal patch. These are generally fabricated using silicon, metals, ceramics, silica glass, carbohydrates, and biodegradable polymers. The drugs are loaded into their reservoir as a solution, microparticulate system, or gels. This system omits the existing shortcomings of the transdermal system, i.e., the poor penetration rate of a drug across the skin, and the advantages eventually add up with benefits such as macromolecular delivery, ease of administration, and painless delivery as the micron needles do not penetrate the dermis where nerve endings are present [188]. The drug is delivered into the dermal layer via a diffusion mechanism [188]. Different penetration strategies are applied for various microneedle approaches, i.e., solid microneedles, coated microneedles, dissolving microneedles, hollow microneedles, and hydrogel microneedles, as shown in Figure 9 [189].

Solid microneedles, or the “poke and patch” approach, use micron-sized insoluble needles to form passages into the skin before applying the drug. The drug diffuses into the deeper layers of skin through these micropores, thereby enhancing permeation [188,190]. Olivia Howells et al. [191] fabricated silicon in-plane solid microneedles using a single wet etch step. Novel microneedles had a characteristic of a 54.7º sidewall etch. The sidewall etching was completed using KOH, forming a sharp pyramidal six-sided tip, effectively enhancing penetration with minimal invasiveness. It is a simple, scalable, and cost-effective method for preparing various micron-sized ranges of solid and hollow microneedles.

Similarly, Tanja Ilic et al. [192] pretreated the skin using stainless steel microneedles to deliver aceclofenac nanoemulsion. The pretreatment improved the bioavailability of aceclofenac by a 1.4- to 2.1-fold increase, thereby enhancing systemic uptake. Coated microneedles, or the “coat and poke” approach, consists of a solid core covered with drug dispersion or solution. The coating thickness depends upon the drug loading. This drug dispersion dissolves within the layers of skin for its action [193]. Zequan Zhou et al. [194] enhanced the delivery efficiency of biopharmaceutics (rhIFNα-1b) complexation-based gel through coated microneedles. The microneedles were prepared by L-polylactide using the micro-moulding method, in which the coating solution was kept at a height of 300 µm. It was observed that rhIFNα-1bgel encapsulated microneedles showed sustained behaviour, besides its better AUC levels and elimination half-life compared to intradermal injection. Coated microneedles also play a vital role in delivering macromolecules such as siRNAs. Wenyi Ruan et al. [195] delivered BRAF siRNA nanocomplexes for anti-melanoma treatment.

The coated needles delivered the peptide efficiently with improved penetrating potential and targeting ability. Dissolving microneedles, or the “poke and release” approach, is a one-step application. Since it comprises, biodegradable polymers containing the drug, it is not withdrawn from the application area; hence, it is used to improve patient compliance. Mengzhen Xing et al. [196] prepared novel dissolving microneedles using biopolymers polyvinyl alcohol and polyvinyl pyrrolidone to treat melasma. The drug, i.e., tranexamic acid, showed a significant drug release, and improved bioavailability and pharmacodynamics compared with solid microneedles and oral administration. Andi Dian Permana et al. [197] showed the effectiveness of intradermal delivery of a lymphatic filariasis drug via dissolving microneedles which enhanced efficiency four-folds and seven-folds higher than oral administration. Hollow microneedles, or the “poke and flow” approach, have hollow chambers inside in which the drugs are loaded. Generally, macromolecular drugs are loaded and deposited into the epidermis or upper dermis layer upon insertion. This type of delivery can monitor the release and flow rate [198]. Vivek Yadav et al. [199] used hollow microneedles to deliver high molecular weight drugs, i.e., rifampicin. The hollow microspheres were prepared via a 3D-printed process of stereolithography technology. Various morphology, ex vivo, and in vivo characterisations revealed that these microneedles have efficient penetration and desired bioavailability.

Generally, microneedles are used to increase the systemic absorption of the drug; however, some studies suggest that microneedles could also be used for targeted skin delivery. A study was conducted to develop an active targeted drug delivery system for the local treatment of HSs. The delivery system consists of a diphenyl carbonate cross-linked cyclodextrin metal-organic framework (CDF) containing more than 26% (*w*/*w*) quercetin (QUE) which was coated with an HSF membrane (QUE@HSF/CDF) and then finally dispersed in Bletilla striata polysaccharide (BSP)-fabricated dissolvable microneedles (BSP-MNs-QUE@HSF/CDF). The results suggested that the biomimetic nano drug delivery system enhanced the therapeutic efficacy of HSs by modulating Wnt/β-catenin and JAK2/STAT3 pathways and decreasing the expression of collagens I and III in HS. Moreover, the authors found that BSP has an additive effect, and the microneedles have greater mechanical strength and improved physical stability than microneedles made of hyaluronic acid. Therefore, the authors concluded that the designed drug delivery system is a promising approach for applications in skin disease treatment and cosmetics [200]. A similar study was conducted by developing pH-sensitive micelles coated with epidermal cells (HaCaT cells) for active targeting of skin diseases. The results indicated that shikonin encapsulated inside the biomimetic nanocarriers accumulated mainly in the active epidermis when delivered with karaya gum-fabricated water-soluble microneedles. The target cells internalised the biomimetic nanocarriers, resulting in swelling and drug release, which increased the therapeutic efficacy of shikonin against imiquimod-induced psoriatic epidermal hyperplasia [201]. 

### Three-Dimensional Printed Microneedles

**Figure 10:**
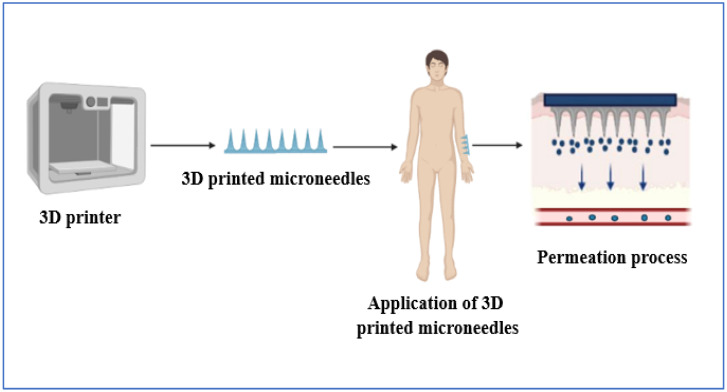
The 3D-printed microneedles for transdermal drug delivery.

Three-dimensional printing or three-dimensional printing technologies have become a versatile method in the design and production of microneedles. This emerging technology tries to address the existing deficiency of traditional methods, thereby making up an ideal process in manufacturing [202]. The manufacturing process includes printing by fused deposition modeling, stereolithography, digital light processing, continuous liquid interface production, and two-photon polymerisation [203,204]. These work by a computer-aided design module, whereby materials are added layer by layer to form a versatile dosage form. They can even form a complex structure of fewer than 0.1 microns in size. The process is rapid and can be utilised for scalability. Various researchers have shown the merits of using 3D printing technology in microneedles, and its diagrammatic representation is shown in Figure 10. Donghyeok Shin and Jinho Hyun et al. [205] prepared silk fibroin microneedles using digital light processing 3D printing technology. The method was opted for because of its simple single-step preparation process and high efficiency for delivering protein, i.e., silk fibroin. Sophia N. Economidou et al. [206] prepared intradermal microneedles using 3D stereolithography technology for insulin delivery. It was observed that 3D-printed microneedles penetrate the skin fast with the minimum application of force. Coated films had strong adhesion on the microneedle surface, improving durability. Md Jasim Uddin et al. [207] fabricated 3D-printed microneedles for cisplatin delivery in cancer treatment. Stereolithography technology results in polymeric microneedles with an 80% penetration capability and an 80–90% release rate. The in vivo activity confirmed high anti-cancer activity and tumor regression.

## 13. Challenges in Transdermal Drug Delivery

Despite progress, numerous problems are yet to be solved, particularly in the enhancement techniques commonly employed for effective TDDSs. To completely understand the penetration mechanism, it is essential to investigate it at the molecular level. In the meantime, barriers related to the cellular level need to be examined to increase retention time and improve delivery efficiency. Moreover, to hasten the translation from bench to bedside, the safety features of the formulation must be assessed methodically at an early stage of the development process. Concerning the future of TDDSs, it is expected that techniques such as transdermal patches and gels will be continuously used to deliver drugs with some specific properties effectively in comparision to oral and parenteral routes. Approaches such as nanoformulations and CPEs will be continuously employed to enhance the transdermal delivery of various drugs. Recently, numerous preclinical studies, clinical trials, and, in some cases, approved and commercialised products have proven the potential of these techniques to deliver drugs via the transdermal route. However, despite having made enormous advancements, successful proof-of-principle studies, and their feasibility in humans, these techniques do not ensure the effective development and commercialisation of a new product. Developing more “cost-effective or delivery-efficient” formulations could be an approach to persuade the distrustful but “off-the-shelf” solutions, primarily developed for “conventional” molecules, which are being used more often nowadays. Little work has been carried out on some drugs by utilising novel drug-designing approaches and formulation optimisation techniques. However, currently, they have gained more attention towards drug delivery via the transdermal route. Formulation development could be a key to the successful exploration of these new technologies in many ways; firstly, it preserves the stability and therapeutic activity of the drug, for example, biotechnology-derived drugs; furthermore, it enables drug partitioning through the skin; and lastly, it provides an efficient drug delivery system that offers targeted delivery of the drug with minimal side-effects. Hence, more efforts need to be focused on the formulation design and development approach so that the potential of novel delivery technologies can be utilised efficiently.

## 14. Conclusions

The skin is an attractive site for the delivery of drugs due to the ease of administration and its large surface area compared to other parts of the body. The transdermal route is one of the most apposite routes for drug delivery. It circumvents the first-pass effect at intestinal and hepatic levels and delivers a constant plasma concentration with negligible fluctuations. The transdermal route’s additional advantages are the ease of access, administration and withdrawal, retentivity, low cost, and high patient compliance. The drug transport mechanism across the skin takes on three main paths: the transcellular (intracellular) route, which entails crossing the cellular membranes with a polar and a lipid domain; the paracellular (intercellular) route, which involves passive diffusion across the extracellular lipid domain; and through the transappendageal routes. When administered by the transdermal route, the major obstacle drugs face is the skin’s barrier properties, specifically the stratum corneum. In an era of cutting-edge technology formulation, scientists working on transdermal drug delivery have made enormous progress in elucidating the exact mechanism of action through DSC, SAXD, WAXD, FT-IR, and confocal Raman spectroscopy. A combination of two CPEs may produce a synergistic effect. However, the mechanistic involvement still needs to be validated. The advancements in synthetic chemistry have opened a broad way to synthesise novel CPEs with enhanced penetration effects, desirable physicochemical properties, and low toxicity levels. In vivo studies are more complicated than in vitro studies due to the skin’s high sensitivity.

## Figures and Tables

**Table 1:** CPEs employed in the transdermal delivery of various drugs along with the mechanism of action.

CPEs	Drug	Mechanism of Action	References
Water	-	Formation of aqueous pore pathway in the stratum corneum	[94,95]
Alcohols	Estradiol Salicylic acid Ibuprofen Levonorgestrel Diclofenac Sodium naproxen Thymoquinone	Extraction of lipids present in between the cells	[97,98,99,100,103,108,109]
Sulfoxides	Fluocinolone acetonide Triamcinolone acetonide Hydrocortisone Testosterone Fluocinonide Bepridil	Distort proteins and modify the intercellular keratin confirmation	[111,112,113,114]
Azones	Cyophenol Naproxen Salicylic acid Celecoxib Thymoquine	Disorganization of well-organized lipid packing of SC bilayer region	[109,119,121,122,123,124]
Surfactants	Ketotifen Lorazepam Diazepam Haloperidol Methyl nicotinate Insulin Hydrocortisone Acyclovir Oestradiol Lactoferrin Lignans	Create a scale-like structure in the lipid phase Swell the SC and interact with the intercellular keratins Lipid fluidization Present as a puddle in the lipid region	[123,127,128,131,133,139,140,141,142,143,144]
Terpenes	5-Fluorouracil and Oestradiol Indomethacin Morphine hydrochloride Imipramine hydrochloride	Modify the solvent nature of the SC and impart partition Interact with intercellular lipids	[147,148,149,150,151]
Pyrrolidone	Melatonin	Lipid fluidization	
Fatty acids	Diclofenac sodium Caffeine	Disorganization of well-organized lipid packing of the SC bilayer region	[159,160]
Phospholipids	Diclofenac	Present as a puddle in the lipid region	[162]
Urea	Indomethacin	Increase the SC water content and act on keratin	[163]
Lipid synthesis inhibitor	Lidocaine Caffeine Levodopa 5-Fluorouracil Insulin	Inhibiting skin lipid metabolism	[164,165,169]
Cell-penetrating Peptides	Cyclosporine A (CsA) Interferon-gamma siRNA	Disrupting the SC lipid structure	[172,174,175]
Ionic liquids	Donepezil Ibuprofen Acyclovir Hydrocortisone and 5-fluorouracil Insulin Hyaluronic acid framework Nucleic acid siRNA	Disruption of cellular integrity, fluidization, and creation of diffusional pathways	[180,181,182,183,184,185,186]

## Data Availability

Not applicable.
